# Dosimeter-Type NO_x_ Sensing Properties of KMnO_4_ and Its Electrical Conductivity during Temperature Programmed Desorption

**DOI:** 10.3390/s130404428

**Published:** 2013-04-02

**Authors:** Andrea Groβ, Michael Kremling, Isabella Marr, David J. Kubinski, Jacobus H. Visser, Harry L. Tuller, Ralf Moos

**Affiliations:** 1 Zentrum für Energietechnik, Bayreuth Engine Research Center (BERC), Department of Functional Materials, University of Bayreuth, 95440 Bayreuth, Germany; E-Mails: andrea.gross@uni-bayreuth.de (A.G.); m.kremling@googlemail.com (M.K); isabella.marr@uni-bayreuth.de (I.M.); 2 Ford Research and Advanced Engineering, Dearborn, MI 48124, USA; E-Mail: dkubinsk@ford.com; 3 Department of Materials Science and Engineering, Massachusetts Institute of Technology, Cambridge, MA 02139, USA; E-Mail: tuller@mit.edu

**Keywords:** NO_x_ dosimeter, lean NO_x_ trap (LNT), precious metal free NO_x_ storage catalyst (NSC), electrical TPD, accumulating sensing principle, low ppm-level NO_x_ detection, *in-situ* catalyst loading state monitoring, ammonia SCR, three-way catalyst (TWC)

## Abstract

An impedimetric NO_x_ dosimeter based on the NO_x_ sorption material KMnO_4_ is proposed. In addition to its application as a low level NO_x_ dosimeter, KMnO_4_ shows potential as a precious metal free lean NO_x_ trap material (LNT) for NO_x_ storage catalysts (NSC) enabling electrical *in-situ* diagnostics. With this dosimeter, low levels of NO and NO_2_ exposure can be detected electrically as instantaneous values at 380 °C by progressive NO_x_ accumulation in the KMnO_4_ based sensitive layer. The linear NO_x_ sensing characteristics are recovered periodically by heating to 650 °C or switching to rich atmospheres. Further insight into the NO_x_ sorption-dependent conductivity of the KMnO_4_-based material is obtained by the novel eTPD method that combines electrical characterization with classical temperature programmed desorption (TPD). The NO_x_ loading amount increases proportionally to the NO_x_ exposure time at sorption temperature. The cumulated NO_x_ exposure, as well as the corresponding NO_x_ loading state, can be detected linearly by electrical means in two modes: (1) time-continuously during the sorption interval including NO_x_ concentration information from the signal derivative or (2) during the short-term thermal NO_x_ release.

## 1. Introduction

Highly sensitive, selective, stable and fast responding NO_x_ sensing devices are required for the reliable detection of low levels of NO_x_ in a number of important application areas, including automotive and industrial emissions control, as well as environmental and air quality monitoring (immission) [1–3]. Often, the main requirement is the ability to monitor NO_x_ mean concentration values over extended periods (e.g., 1-hour value for immission legislation [4], or the emitted concentration per driven distance [5]) instead of the instantaneous concentration. Dosimeter, integrating or accumulating-type sensors, largely operated as optical or mass sensitive devices, are designed to meet these requirements. Analyte accumulation affects the sensor signal and is achieved either by the generation of a reaction product with the sensor active layer [6–11], or irreversible sorption of NO_x_ onto the surface layer [12–14], followed by periodic regeneration of the sorption capacity [12,14–17].

Recently, impedimetric or resistive NO_x_ dosimeters, based on materials utilized in automotive lean NO_x_ trap catalysts (LNT), were successfully introduced [6,11,18]. Around 400 °C, these carbonate-based materials enable long-term detection of low levels of NO and NO_2_ by monitoring the increase in conductivity with increased NO_x_ loading. The zero-level is reset by regeneration, achieved either by a step change in temperature or by exposure to reducing atmospheres [6].

In this study, KMnO_4_ is investigated as a low-cost alternative to commercial LNT formulations in a dosimeter-type NO_x_ sensing device. KMnO_4_ is known as a strong oxidant [19–21], forming nitrites and nitrates upon exposure to NO_x_, even above the KMnO_4_ decomposition temperature [22,23]. Following an investigation of its electrical properties, the NO_x_ dosimeter-type sensing properties at elevated temperatures and the effect of periodic thermal regeneration are examined. The NO_x_ dose is measured either during sorption or during regeneration by combining the conventional temperature programmed desorption method with the electrical sensor signal. This technique, denoted as eTPD, provides, for the first time, a quantitative correlation between the electrical properties and the NO_x_ loading state of a material. This should be of interest for both sensing and catalyst diagnosis applications.

## 2. KMnO_4_/La-Al_2_O_3_ as Sensitive Layer

### 2.1. Sample Preparation and Characterization

**Figure 1.**
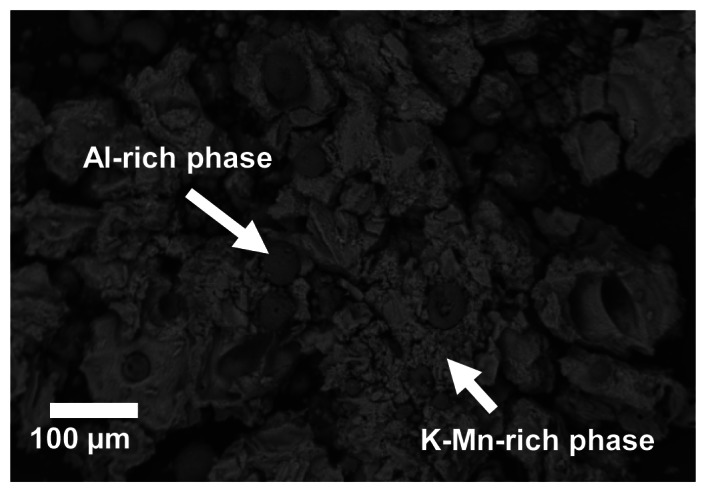
SEM image (BSE) of KMnO_4_/La-Al_2_O_3_ powder after firing. The Al-rich particles and the K-Mn-rich matrix are indicated.

The sensitive layer of the proposed NO_x_ dosimeter was prepared from 17 mol% KMnO_4_ (Merck) deposited onto alumina, stabilized with 3% lanthanum (Puralox SCFa-140La3), serving as support oxide with surface area of 140 m^2^/g and mean particle diameter of 30 μm. The KMnO_4_/La-Al_2_O_3_ powder was prepared by multiple infiltration of an aqueous solution of KMnO_4_ into the La-Al_2_O_3_ powder, followed by drying at 100 °C and calcination at 600 °C for 5 h. Upon thermal decomposition KMnO_4_ is known to form various potassium- and manganese-containing compounds, like K_2_MnO_4_ and K_3_MnO_4_, as well as manganese oxide MnO_x_ existing in different oxidation states [22,24–30]. The decomposition of KMnO_4_ is reported by Boldyrev [25,26] to become noticeable in the temperature range from 205 to 280 °C. KMnO_4_-impregnation and firing decreased the surface area of the powder to 100 m^2^/g (obtained by BET method (named after the originators of the method: Brunauer, Emmett, and Teller) relying on the adsorption of gases to determine the specific surface area of powders). The SEM (scanning electron microscope) analysis of the fired KMnO_4_/La-Al_2_O_3_ powder is given in Figure 1 as a backscatter electron (BSE) image. The powder consists of spherical La-Al_2_O_3_-rich particles in the range of some tens of μm, partly embedded in a potassium and manganese comprising matrix, which was confirmed by energy-dispersive X-ray spectroscopy (EDX) measurements.

The KMnO_4_/La-Al_2_O_3_ powder was mixed with an organic binder (KD2721, Zschimmer & Schwarz) in order to obtain a processable paste. The paste was deposited by spatula onto a 96% pure alumina substrate equipped with gold interdigitated electrodes (area 5 × 6 mm, finger width/distance 100 μm) and fired at 600 °C. The sample was pre-conditioned for several hours at temperatures up to 650 °C in NO_x_ containing oxygen-rich atmospheres.

**Figure 2.**
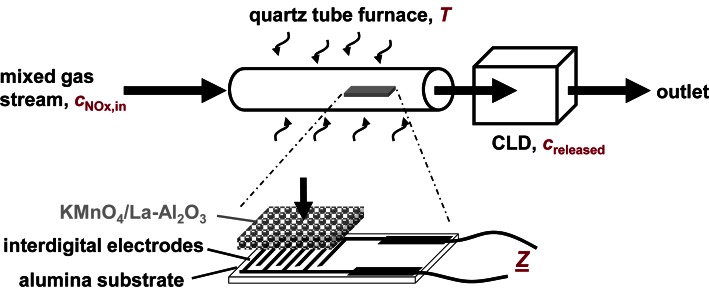
Sensor setup and test apparatus including the gas dosing system, a quartz tube furnace containing the KMnO_4_/La-Al_2_O_3_ sample and a chemiluminescence detector (CLD, 700 EL ht, Ecophysics).

The electrical properties of KMnO_4_/La-Al_2_O_3_ were investigated in a test apparatus as sketched in Figure 2. Following installation in a quartz-tube furnace with inner diameter of 22 mm, the sample was heated to temperatures between 300 to 650 °C. The KMnO_4_/La-Al_2_O_3_ layer was exposed to a 2 L/min lean gas flow (10% O_2_, 50% N_2_ humidified with a water bubbler at room temperature, and 5% CO_2_ diluted in N_2_ balance) with a gas exchange time of the system in the range of 8 s. The impedance *Ẕ* of the KMnO_4_/La-Al_2_O_3_ sample was recorded by an impedance analyzer (Alpha High Performance Frequency Analyzer, Novocontrol).

### 2.2. Electrical Properties in the Unloaded State

The electrical properties of KMnO_4_/La-Al_2_O_3_ in the unloaded state (after regeneration of the NO_x_ sorption sites) were evaluated from 300 to 650 °C by impedance spectroscopy in the frequency range of 1 Hz to 1 MHz. Plotting the impedances in the complex plane as Nyquist plots (real part *Z′* and imaginary part *Z″*) yields near semicircular spectra at higher frequencies. This allows the bulk impedance to be modeled by a resistance *R* in parallel to a constant phase element *CPE* (*R*‖*CPE*). The corresponding impedance *Ẕ*_CPE_ expressed as a function of the model parameters *n* (ranging from 0 to 1) and *Q* as well as the angular frequency *ω* is given in Equation (1):

(1) $${\underline{Z}}_{\text{CPE}}(\omega )=\frac{1}{Q}{\left(\text{i}\omega \right)}^{-n}$$

**Figure 3.**
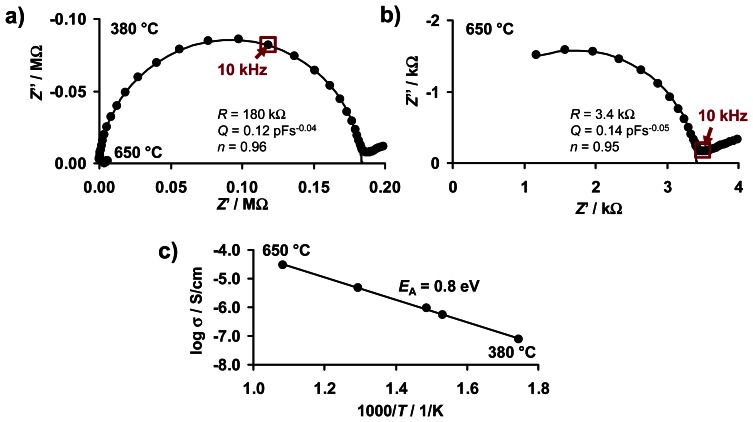
Thermal activated conductivity of KMnO_4_/La-Al_2_O_3_: (**a**) Nyquist plot of the impedance Ẕ at 380 and 650 °C, (**b**) enlargement of 650 °C data, (**c**) Arrhenius-like representation of conductivity σ from 300 to 650 °C. Further time-continuous impedance measurements were conducted at 10 kHz (see Section 3.1) and the 10 kHz data points in (**a**) and (**b**) are highlighted.

In Figure 3(a and b), examples for the corresponding Nyquist plots of the measured impedance data (dots) for 380 and 650 °C, together with the fitted *R*‖*CPE* curves in the upper frequency range (solid curves) and the corresponding fitting parameters, are displayed. While the KMnO_4_/La-Al_2_O_3_ sample has a resistance of 180 kΩ at 380 °C, it decreases to 3.4 kΩ upon heating to 650 °C.

The electrical conductivity σ of the KMnO_4_/La-Al_2_O_3_ specimen was estimated from the fitted *R*-values of the impedance in the *R*‖*C* dominated frequency range taking into account the electrode geometry. The electrode geometry is estimated from the capacitance of the uncoated structure, assuming a parallel-plate capacitor, as described in [31]. The resulting Arrhenius-like representation of σ as a function of inverse temperature 1/*T* in Figure 3(c) gives a thermal activation energy of the conductivity *E*_A_ of 0.8 ± 0.1 eV. This thermally activated conductivity leads to an almost two decades increase from ∼5·10^−7^ S/cm at 380 °C to 3·10^−5^ S/cm at 650 °C.

Information regarding the electrical conductivity of KMnO_4_-based materials above the decomposition temperature in the literature is limited. In thermoelectric tests, KMnO_4_ and its decomposition products were identified as *n*-type semiconductors by Boldyrev and Kabanov, and in the literature cited therein [26,28]. Upon thermal decomposition, the conductivity of KMnO_4_ was found to increase, and depending on morphology, the conductivity was reported to range from 10^−6^ to 10^−8^ S/cm at 170 to 210 °C [26,28].

The lower conductivity of the KMnO_4_/La-Al_2_O_3_ based material under investigation compared to the reported conductivity of pure KMnO_4_ is attributed to the less conductive La-Al_2_O_3_ particles serving as support oxide in the applied sensitive coating. Recently published results on the K_2_CO_3_/La-Al_2_O_3_ system indicate a significant contribution of La-Al_2_O_3_ to the measured conductivity given its lower conductivity than that of pure K_2_CO_3_ [32].

## 3. NO_x_ Sensing Properties

Similar to passive samplers, dosimeter-type gas sensors are operated in two alternating steps: Analyte molecules are progressively accumulated in the sensitive layer during a sorption period, followed by a regeneration procedure to release the formerly sorbed molecules. The focus of the next section is on the evaluation of the dosimeter-type NO_x_ sensing characteristics of KMnO_4_/La-Al_2_O_3_ during NO_x_ sorption as well as the efficiency of thermal regeneration.

### 3.1. Experimental Setup and Data Evaluation

To study the effect of NO_x_ in the low ppm range, the KMnO_4_/La-Al_2_O_3_ sample was exposed to various NO and NO_2_ concentrations, *c*_NO,in_ and *c*_NO2,in_ for defined time intervals *t*_NOx,in_. NO_x_ was admixed to the 2 L/min lean base gas flow (10% O_2_, 50% N_2_ humidified with a water bubbler at room temperature, and 5% CO_2_ diluted in N_2_ balance). The outlet concentrations were determined by a chemiluminescence detector, as illustrated in Figure 2. In accordance to the reported catalytic activity of Mn-containing LNTs [23,33–36], as well as to results on LNT-based NO_x_ dosimeters [6,18], the NO_x_ sorption studies were performed at a sorption temperature *T*_sorption_ of 380 °C, with periodic heating to 650 °C for regeneration.

The sample impedance was recorded continuously during NO_x_ exposure. Since the fitted *n*-parameters of Ẕ_CPE_ (Equation (1)) of KMnO_4_/La-Al_2_O_3_ in the high frequency range were found to be close to 1 (≈0.95), *Q* can be approximated by the capacitance *C* and the *R*‖*CPE* equivalent circuit model can be simplified to an *R*‖*C* circuit. Thus, *R* is calculated from the absolute value of the impedance |*Ẕ*| and the phase angle *φ* at a fixed frequency according to Equation (2).

From the Nyquist plots in Figure 3, 10 kHz (marked in red) was selected as an appropriate measurement frequency to monitor the temperature dependent electrical properties in the *R*‖*C* dominated range over time and was used, if not denoted otherwise. The absolute value of the relative resistance change due to NO_x_ exposure Δ*R*_rel_ is denoted as the sensor signal, and is defined by Equation (3), with *R*_0_ being the base resistance in the NO_x_ unloaded state:

(2) $$R=\left|Z\right|\cdot \sqrt{1+\tan^{2}\varphi }$$

(3) $$\Delta R_{\text{rel}}=\frac{\left|\Delta R\right|}{R_{0}}=\frac{R_{0}-R}{R_{0}}$$

**Figure 4.**
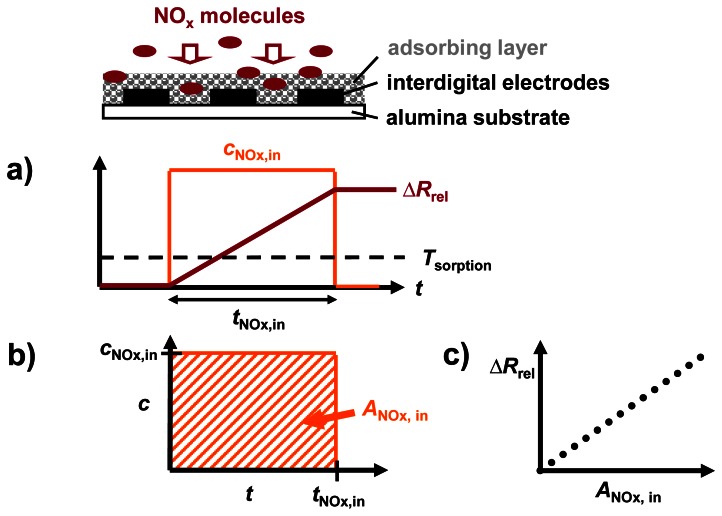
Data analysis during progressive NO_x_ accumulation at *T*_sorption_: (**a**) increase in sensor response Δ*R*_rel_ during NO_x_ sorption, (**b**) determination of cumulated NO_x_ exposure *A*_NOx,in_, (**c**) characteristic Δ*R*_rel_*vs. A*_NOx,in_ line.

The analysis in terms of dosimeter-type sensing properties during NO_x_ sorption at constant flow rates is illustrated in Figure 4. During the NO_x_ loading stage, Δ*R*_rel_ is expected to increase in the presence of NO_x_ at *T*_sorption_ due to progressive NO_x_ accumulation, without recovery (Figure 4(a)). In the case of a constant flow rate, the cumulated NO_x_ exposure (or dose) *A*_NOx,in_ is given by the time integral of *c*_NOx,in_ as sketched in Figure 4(b), resulting in the unit ppm·s [6,16,37].

At a constant NO_x_ concentration, *A*_NOx,in_ scales linearly with *t*_NOx,in_. The resulting characteristic line in Figure 4(c) correlates Δ*R*_rel_ with *A*_NOx,in_. It has been shown in detail for a similar material in [6] that in the case of a linear correlation, the signal derivative of a NO_x_ dosimeter at a constant flow rate increases with the actual NO_x_ concentration.

### 3.2. Cumulative NO_x_ Detection at 380 °C

**Figure 5.**
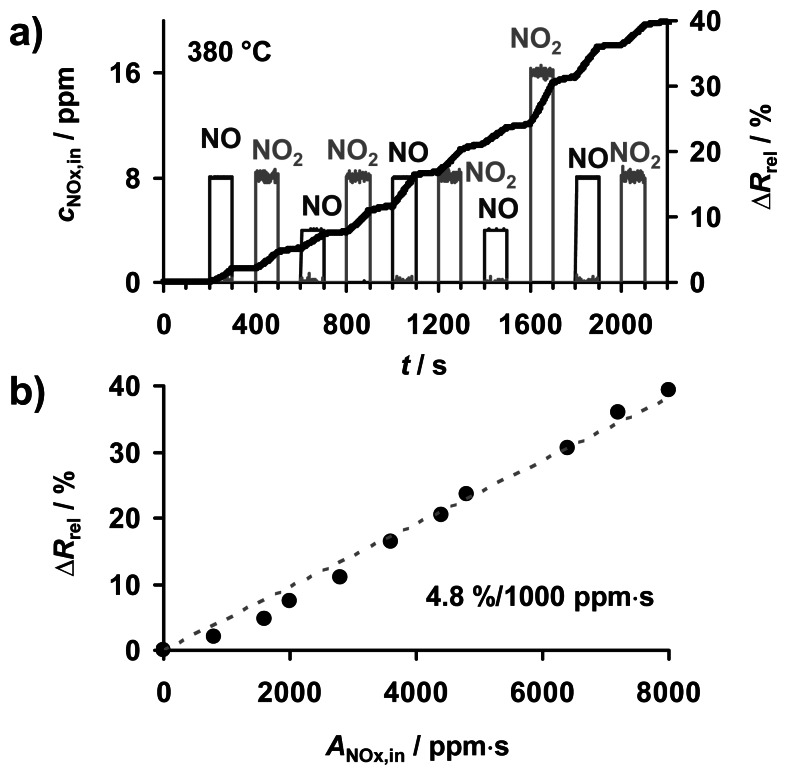
NO_x_ sensing properties at 380 °C (10% O_2_, 50% N_2_/H_2_O, 5% CO_2_ in N_2_): (**a**) stepwise increase of sensor response Δ*R*_rel_ (Equation (3)) during cyclic exposure to NO or NO_2_, (**b**) resulting linear Δ*R*_rel_*vs. A*_NOx,in_ characteristic line.

The presence of NO_x_ was found to decrease the resistivity of KMnO_4_/La-Al_2_O_3_, with the electrical response continuing to satisfy the *R*‖*C* equivalent circuit (not shown). The temporal dependence of *R* on NO_x_ at 380 °C was studied by exposing the sample to pulses of NO and NO_2_ for periods of *t*_NOx,in_ = 100 s with concentrations ranging up to 16 ppm. The pulse heights in terms of *c*_NO,in_ and *c*_NO2,in_, together with the resulting sensor response, are displayed in Figure 5(a). The sensor response Δ*R*_rel_ (Equation (3)) increases stepwise in the presence of NO and NO_2_ without any recovery at 0 ppm NO_x_. The slope of Δ*R*_rel_*vs. t* increases with *c*_NOx,in_. The characteristic line in Figure 5(b) is extracted from the measured data points and the course of the NO_x_ concentration according to Figure 4. Δ*R*_rel_ correlates almost linearly with the cumulated NO_x_ exposure *A*_NOx,in_, independent of the NO_x_ species, up to at least 40% signal change with a NO_x_ sensitivity of 4.8%/1,000 ppm·s. The specimen thus provides comparable sensitivity to both NO and NO_2_. Small deviations of the NO_2_ related data points from linearity at the initial stage of exposure may originate from NO_2_ adsorption on the inner surface of the feed lines. The sensing characteristics and in particularly the sensitivity of NO_x_ dosimeter with a comparable sensitive material were found to be dependent on the temperature as well as on the thickness of the sensitive layer [6,16].

Figure 5 indicates the strong and progressive sorption of NO and NO_2_ onto KMnO_4_/La-Al_2_O_3_ at 380 °C with corresponding impact on its electrical conductivity. As in K-Mn-containing LNTs [21,23], NO_x_ is expected accumulate on KMnO_4_/La-Al_2_O_3_ by forming stable nitrates on the potassium sites generated upon KMnO_4_ decomposition [22]. For LNTs it is well known that NO is first oxidized to NO_2_ on redox active sites provided by e.g., precious metals, followed by chemical NO_2_ storage by reaction with the alkaline (earth-) carbonates, mainly BaCO_3_ or K_2_CO_3_, to form nitrates [38–40]. The observed increase in the conductivity of fully formulated LNTs in NO_x_ enables their application as total NO_x_ sensors [6,11,41] or for *in-situ* diagnostics of automotive catalysts [41–43].

The requirement of an incorporated oxidant, for the purpose of NO sorption, was verified by electrical means. Pure BaCO_3_ or K_2_CO_3_, on the other hand, accumulates only NO_2_, enabling conductometric NO_2_ dosimetry, without NO cross-sensitivity [15,32]. Given the ability of KMnO_4_/La-Al_2_O_3_ to detect either NO or NO_2_, the oxidizing properties of KMnO_4_/La-Al_2_O_3_ are demonstrated to be sufficient to convert NO to NO_2_ prior to nitrate formation. This is consistent with MnO_x_, as a product of KMnO_4_ decomposition [25,27], being known as an effective oxidizing agent in NO_x_ reduction catalysts [22,24,33,34,36,44]. The contribution of MnO_x_ to the NO_x_ sorption capacity at 380 °C cannot be excluded [45–48].

The linear correlation between Δ*R*_rel_ and *A*_NOx,in_ in the low loading state of the NO_x_ dosimeter based on KMnO_4_/La-Al_2_O_3_ in Figure 5 points on a sorption rate proportional to the NO_x_ concentration. This linearity provides a dual-mode functionality: while the sensor response corresponds directly to the cumulated NO_x_ exposure during the sorption period, the course of *c*_NOx,in_ can be determined via the signal derivative as described in [6,14]. Furthermore, these results demonstrate that decomposed KMnO_4_ can be utilized in NO_x_ dosimeters and catalysts *without* any need for expensive precious metal additives due to its intrinsic oxidizing nature.

### 3.3. NO_x_ Concentration Sensitivity at 650 °C

An important criterion for a useful sensor is the ability to refresh or regenerate the device following accumulation of the target gas analyte, which in this study is NO_x_. The decreased thermodynamic stability of the formed nitrates upon heating limits the catalytic activity of LNTs [38–40,49] and alters the cumulative NO_x_ sensing characteristics of carbonates and LNT-based sensors [6,17,18,32]. According to Becerra et al.[22], nitrate and nitrite-like compounds formed on KMnO_4_-based materials decompose in the temperature range of ∼550 to 670 °C. Hence, a thermal release of sorbed NO_x_, leading to a recovery of the sorption sites of KMnO_4_/La-Al_2_O_3_, seems feasible.

**Figure 6.**
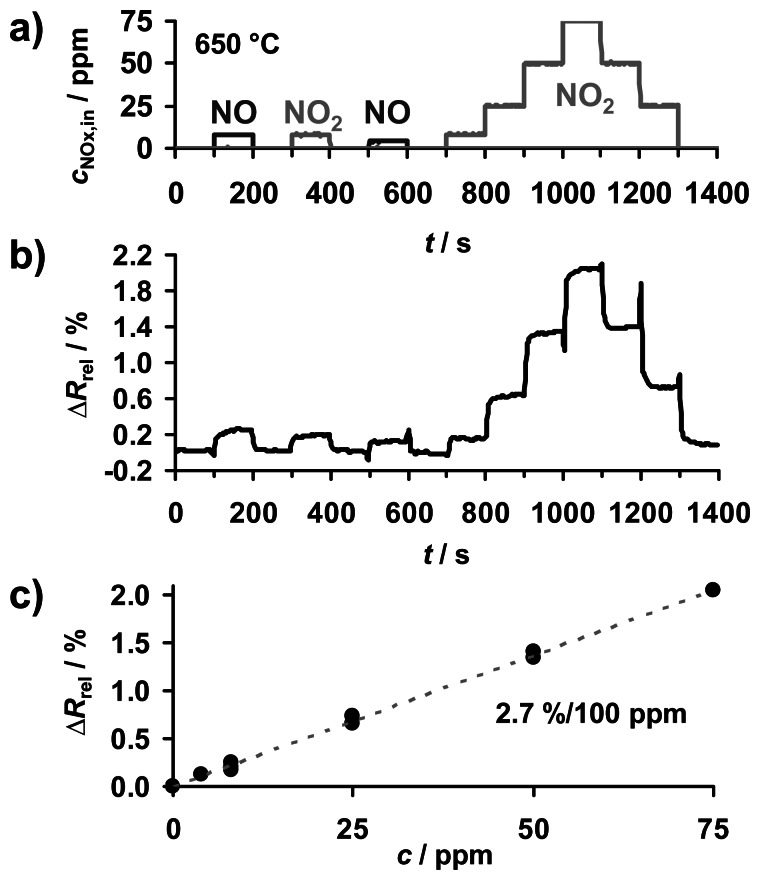
NO_x_ concentration detection at 650 °C: (**a**) course of NO_x_ concentration *c*_NOx,in_, (**b**) sensor response Δ*R*_rel_, (**c**) linear correlation between Δ*R*_rel_ and *c*_NOx,in_.

The effect of NO_x_ on the resistivity of KMnO_4_/La-Al_2_O_3_ was studied at 650 °C to investigate this temperature as being suitable for regeneration. The sample was exposed to the NO_x_ concentration profile shown in Figure 6(a) with up to 8 ppm NO and 75 ppm NO_2_. Δ*R*_rel_ is calculated from the impedance at 1 MHz due to the increased conductivity. Again, the conductivity of KMnO_4_/La-Al_2_O_3_ increases in the presence of NO_x_. But, as shown in Figure 6(b), at 650 °C, the value for Δ*R*_rel_ follows the course of *c*_NOx,in_ (instead of ∫*c*_NOx,in_ d*t*) being characteristic of a common concentration-detecting gas sensor response. Despite the corresponding concentration-related characteristic line in Figure 6(c), which gives a linear correlation, the low sensitivity of only 2.7%/100 ppm NO_2_ limits the application of KMnO_4_/La-Al_2_O_3_ as a NO_x_ sensing material operated at 650 °C.

The reversibly sensor response at 650 °C in Figure 6 indicates that the equilibrium of the NO_x_ sorption on the KMnO_4_-based material is shifted to the side of the reactants and the resulting fast desorption goes along with the loss of NO_x_ accumulation capability. Hence, 650 °C seems an appropriate temperature to release formerly sorbed NO_x_ and to recover the sorption capacity, as well as the electrical properties of KMnO_4_/La-Al_2_O_3_. The reversibility of the sensor response of KMnO_4_/La-Al_2_O_3_ at 650 °C is consistent with results on an LNT-based NO_x_ dosimeter [17].

### 3.4. Efficiency of Thermal Regeneration

**Figure 7.**
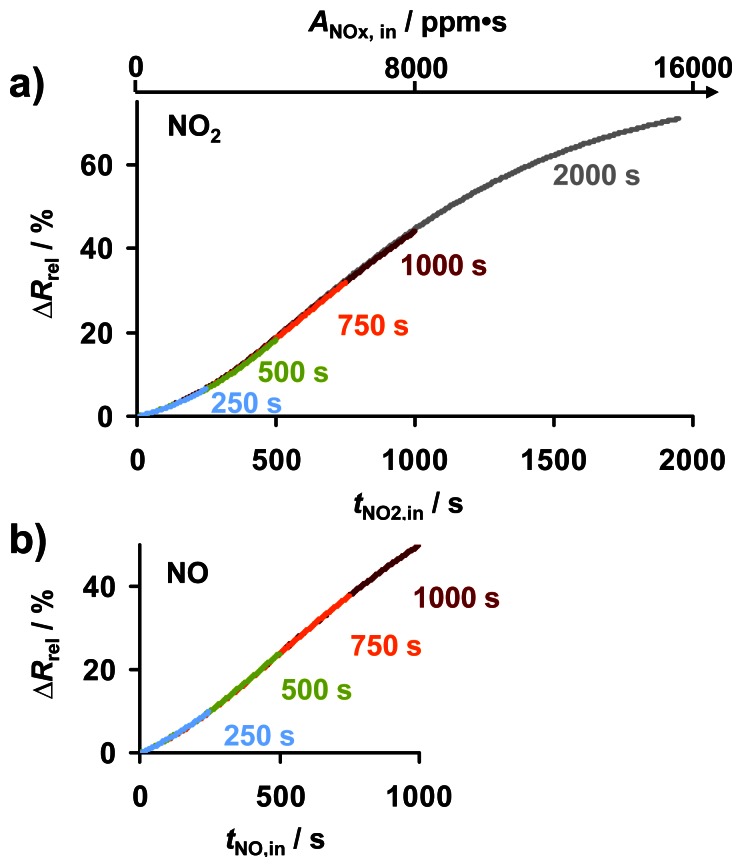
Repeated sensor response to 8 ppm NO_x_ intervals with intermediate regeneration: (**a**) sensor response Δ*R*_rel_ during NO_2_ for NO_2_ exposure of *t*_NO2,in_ as indicated, (**b**) Δ*R*_rel_ during NO for *t*_NO,in_ as indicated.

In the following test series, the efficiency of thermal regeneration, the reproducibility of the dosimeter-type NO_x_ sensing characteristics, and the influence of NO_x_ exposure time were studied. The same KMnO_4_/La-Al_2_O_3_ sample was exposed to 8 ppm NO_2_ or NO at 380 °C in periods of 250, 500, 750, 1,000, and 2,000 s. Between each NO_x_ exposure period, the sample was regenerated at 650 °C for about 5 min in the lean gas flow. The sensor responses as a function of *t*_NO2,in_ and *t*_NO,in_ are compared in Figure 7. The five NO_2_-borne curves of Δ*R*_rel_ depicted in Figure 7(a) are almost identical in the corresponding overlapping time scales; please note that the data points corresponding to the longest NO_2_ exposure of 2,000 s are partly masked by the other data curves. The corresponding NO curves up to 1,000 s in Figure 7(b) are overlapping as well. The sensor behaves linearly (following an initial incubation period) up to a resistance change of about 40%, with the slope of Δ*R*_rel_ in Figure 7(a) being nearly constant up to about 1,000 s (8,000 ppm·s NO_2_). The nonlinearity at the beginning of NO_x_ exposure in Figures 5 and 7, *i.e.*, the slight initial slope increase during the first 375 s in NO_2_ (3,000 ppm·s NO_2_), is assumed to be caused by NO_x_ (in particular NO_2_) being adsorbed on the feed gas lines resulting in a delayed sensor response. Further NO_2_ exposure leads to a decrease in the slope and Δ*R*_rel_ reaches a value of 70% after half an hour in 8 ppm NO_2_. By definition, Δ*R*_rel_ cannot reach 100% as the conductivity increases, resulting in a flattening of the curve of Δ*R*_rel_ with continuing NO_x_ loading. It is expected that a greater sensitive layer thickness would increase the linear range to higher NO_x_ levels, but at reduced sensitivity (slope d(Δ*R*_rel_)/d*A*_NOx,in_), as reported for LNT-based dosimeters [16].

The reproducibility of the sensor response in Figure 7 indicates that the sorption sites of the KMnO_4_/La-Al_2_O_3_-based dosimeter material can be recovered by releasing sorbed NO_x_ thermally. Heating up to 650 °C restores the NO_x_ sensing characteristics at 380 °C, independent of the former NO_x_ exposure duration. The base resistance in the unloaded state *R*_0_ was found to decrease slightly with time without impacting the NO_x_ sensitivity. This might be attributed to small morphological changes during thermal aging, which, however, are too small to be seen by SEM. It is noteworthy to mention that the dosimeter principle avoids such long term signal drifts by definition, since the zero level of Δ*R*_rel_ is reset after each regeneration step. This is one of the key advantages of conductometric dosimeters compared to classical semiconductor gas sensors.

## 4. Electrical Conductivity during Temperature Programmed Desorption (eTPD)

As in temperature programmed desorption (TPD) studies, the course of the NO_x_ concentration due to NO_x_ desorption during thermal regeneration gives quantitative information about the amount of stored NO_x_ in KMnO_4_/La-Al_2_O_3_. Combining TPD with simultaneous electrical characterization (eTPD), an electrical readout of the cumulative sorbed NO_x_ during the short thermal regeneration periods results. To obtain further insight into the relation between the NO_x_ loading state and the electrical behavior during NO_x_ sorption and release, eTPD is applied to the KMnO_4_/La-Al_2_O_3_ formulation.

### 4.1. eTPD Setup and Data Evaluation

**Figure 8.**
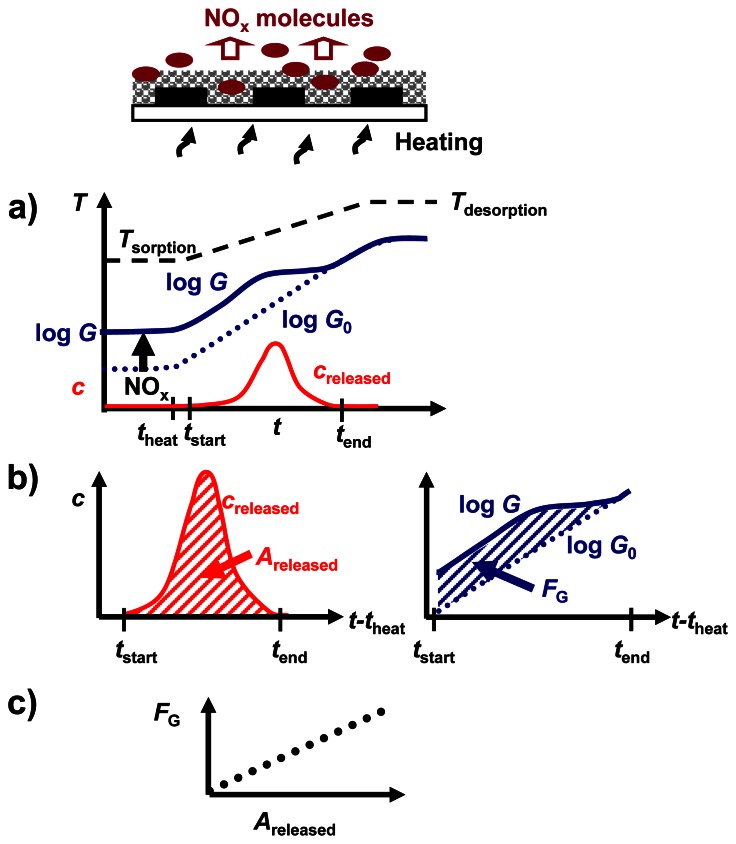
Data analysis for eTPD: (**a**) time dependence of conductance log *G* and outlet NO_x_ concentration *c*_released_, (**b**) determination of released amount *A*_released_ and electrical response *F*_G_, (**c**) *F*_G_ as a function of *A*_released_.

eTPD on KMnO_4_/La-Al_2_O_3_ is performed with the experimental arrangement shown in Figure 2. The eTPD related data and their evaluation are summarized in Figure 8.

To recover the NO_x_ sensing characteristics of KMnO_4_/La-Al_2_O_3_ in between the NO_x_ sorption intervals in the lean gas flow in Figure 7, the sample was heated from 380 °C (*T*_sorption_) to 650 °C (*T*_desorption_) with a heating rate of 74 °C/min from 425 to 635 °C, while monitoring the impedance at 10 kHz. The temperature increase started at *t*_heat_, 50 s after the end of the preceding NO_x_ dosing interval. The resulting NO_x_ desorption curve (Figure 8(a)) is displayed as a NO_x_ concentration *c*_released_. At *t*_start_ = *t*_heat_ + 50 s an increase of NO_x_ is observed in the outlet until *t*_end_. As illustrated in Figure 8(b), at a constant flow rate, the time integral of *c*_released_, evaluated in the time interval of *t*_end_ – *t*_start_ = 300 s, reflects the released NO_x_ amount *A*_released_. *A*_released_ is expected to be proportional to the quantity of sorbed NO_x_, if the sorption sites are fully recovered by heating.

The conductance *G* of KMnO_4_/La-Al_2_O_3_, with *G* = 1/*R*, was found to be affected by the temperature and the NO_x_ loading level, both changing during thermal regeneration. As sketched in Figure 8(a), NO_x_ release results in a convergence of *G* to *G*_0_ = 1/*R*_0_, *G*_0_ being the temperature dependent conductance in the unloaded state. The time integral of the conductance upon heating, relative to those of *G*_0_, is evaluated as the cumulative electrical response *F*_G_. *F*_G_ is calculated according to Equation (4) and is shown in Figure 8(b) as the area between the curves corresponding to the two loading states. In the ideal case, *F*_G_ would be a measurand for *A*_released_ (Figure 8(c)) and would depend linearly on the cumulative sorbed NO_x_ amount.

(4) $$F_{\text{G}}=\int_{t_{\text{start}}}^{t_{\text{end}}}[\log G(t)-\log G_{0}(t)]\text{d}t$$

### 4.2. Evaluation of the Released Amount

**Figure 9.**
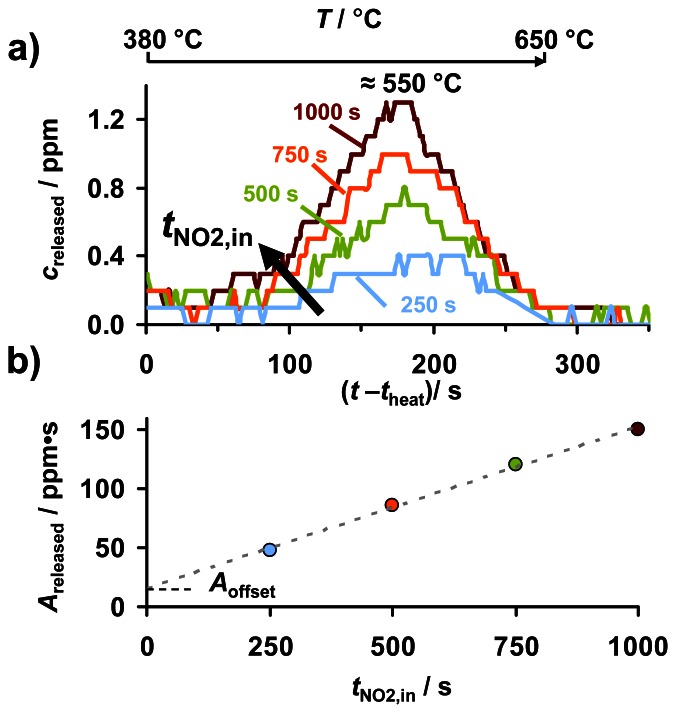
NO_x_ release during heating to 650 °C after 8 ppm NO_2_ exposure for 250 s, 500 s, 750 s, and 1,000 s: (**a**) outlet NO_x_ concentration *c*_released_, (**b**) area *A*_released_ below the curve as depicted in Figure 8(b) as a function of NO_2_ loading time *t*_NO2,in_.

In Figure 7, the resistance responses of KMnO_4_/La-Al_2_O_3_ during an NO_2_ sorption series at 380 °C with various exposure periods *t*_NO2,in_ are reported. The subsequent regeneration by heating to 650 °C to release the formerly sorbed NO_x_ can be analyzed in terms of TPD. The corresponding outlet NO_x_ concentrations *c*_released_ in the lean 2 L/min gas flow, with a resolution of 0.1 ppm given by the CLD, for *t*_NO2,in_ up to 1,000 s are compared in Figure 9. After 1,000 s in 8 ppm NO_2_ (red curve, as indicated in Figure 9), KMnO_4_/La-Al_2_O_3_ starts to release NO_x_ at about 400 °C. *c*_released_ increases with temperature, and at about 550 °C, a maximum is reached at about 1.3 ppm. Shorter NO_2_ exposure periods, representing a lower amount of NO_x_ loading, yield lower peak heights of *c*_released_. At 650 °C, *c*_released_ reaches zero for all curves, indicating the end of NO_x_ release. Additionally, both peak maximum and desorption onset are shifted to lower temperatures with increasing *t*_NO2,in_. The latter points to a lower stability of the sorbed NO_x_ with increased NO_x_ loading. Concerning the low values of *c*_released_, it should be considered that the evolved NO_x_ is diluted in the 2 L/min lean gas flow and that the sensitive KMnO_4_ coating amounts only to an area of about 30 mm^2^ (5 × 6 mm).

The reproducibility of the dosimeter-type NO_x_ sensing characteristics at 380 °C (Figure 7) and the missing NO_x_ accumulation at 650 °C (Figure 6) reveal that the NO_x_ sorption capacity of KMnO_4_/La-Al_2_O_3_ can be recovered by heating to 650 °C. Therefore, the quantity of sorbed NO_2_ on KMnO_4_/La-Al_2_O_3_ can be estimated from the subsequently thermally released NO_x_ amount *A*_released_, being the area under the desorption peak as shown in Figure 8(b). Figure 9(b) reveals that *A*_released_ and hence the amount of NO_x_ sorbed in KMnO_4_/La-Al_2_O_3_ increases almost linearly (with only a small offset) with NO_2_ exposure, reflected by *t*_NO2,in_. Consequently, NO_2_ is sorbed on KMnO_4_/La-Al_2_O_3_ with a time constant sorption rate during the 8 ppm NO_2_ exposure periods. After 1,000 s in 8 ppm NO_2_, resulting in a cumulated NO_2_ exposure of 8,000 ppm·s, about 150 ppm·s NO_x_ are released. This indicates that only about 1.9% of the NO_2_ in the passing gas flow is sorbed in the KMnO_4_ based sensitive layer. The gas velocity of 5.3 m/min together the sensitive area length of 6 mm amounts to a residence time of about 70 ms, being comparable to those in catalysts [37]. However, the huge gas volume above the sensitive layer in the 22 mm diameter quartz tube inhibits full NO_x_ storage in this NO_x_ dosimeter setup. The small offset of *A*_released_ in Figure 9(b) amounts to about *A*_offset_ ≈ 15 ppm·s. If one divides this value by the integration time of 300 s, one obtains an average concentration of 0.05 ppm NO_x_. A closer look at Figure 9(a) reveals that this is (roughly) the offset of the NO_x_ concentration measurement by the CLD with a resolution of 0.1 ppm. As a conclusion, the offset of *A*_released_ can be attributed to an integration error. The analysis of the corresponding data after NO exposure yield the same qualitative results (data not shown here) but with a smaller offset. Hence, a further explanation might be the partial overlap of *c*_released_ with the preceding decay of *c*_NO2,in_ due to NO_2_ adsorption in the feed lines.

**Figure 10.**
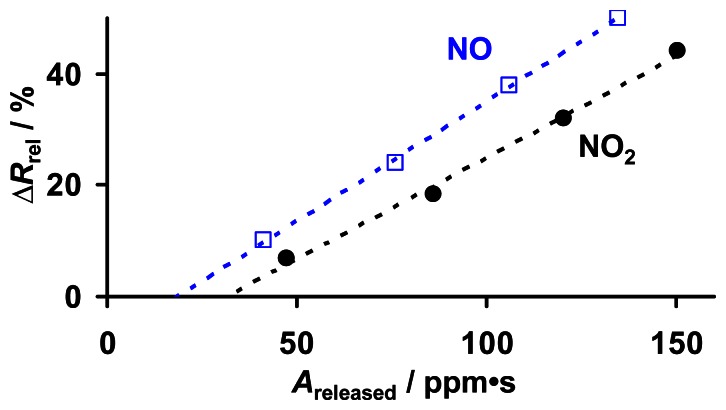
Correlation between the sensor response Δ*R*_rel_ during NO and NO_2_ sorption and the NO_x_ amount *A*_released_ obtained from subsequent TPD.

The observed sensor response Δ*R*_rel_ of KMnO_4_/La-Al_2_O_3_ during NO_x_ sorption at 380 °C (Figure 7) obviously corresponds to the amount of loaded NO_x_. In Figure 10, Δ*R*_rel_, caused by 8 ppm NO or NO_2_ for up to 1,000 s, is related to *A*_released_, which is obtained from the subsequent regeneration shown in Figure 9. For NO exposure, as well as for NO_2_ exposure, Δ*R*_rel_ increases linearly with *A*_released_. Hence, in the investigated range, Δ*R*_rel_ serves as a linear measure for the NO and NO_2_ loading levels of KMnO_4_/La-Al_2_O_3_, and due to the constant NO_x_ sorption rate (Figure 9(b)), also for the cumulated NO_x_ exposure (NO_x_ dose). Thereby, the conductivity of KMnO_4_/La-Al_2_O_3_ is slightly more sensitive to NO compared to NO_2_. From a catalytic point of view, it would be expected that NO_2_ in the gas flow influences the material's properties more than NO, since NO_2_ can be sorbed directly as nitrate, whereas NO needs to be oxidized first [23,36]. However, the manganese oxide components of the decomposed KMnO_4_ might become reduced upon oxidizing NO, thereby affecting the conductivity of KMnO_4_/La-Al_2_O_3_ and hence the NO sensitivity. The delay in the sensor response resulting in an x-axis intercept in Figure 10 is expected to be caused by the already discussed inaccuracy of the determination of *A*_released_ by integration of small values of evolved NO_x_ (*A*_Offset_ ≈ 15 ppm·s in Figure 9(b)). In addition, NO_x_ (in particular NO_2_) adsorption to the feed lines lowers the sensor response but increases the analyzed value for the desorbed amount.

Combining the classical TPD method, with the dosimeter-type electrical response of KMnO_4_/La-Al_2_O_3_, demonstrates the possibility of sensing NO_x_ exposure and of electrically monitoring the NO_x_ loading level of the NO_x_ sorbent *in-situ*, both with linear correlation at low loading.

### 4.3. Electrical Information upon Thermal Regeneration

**Figure 11.**
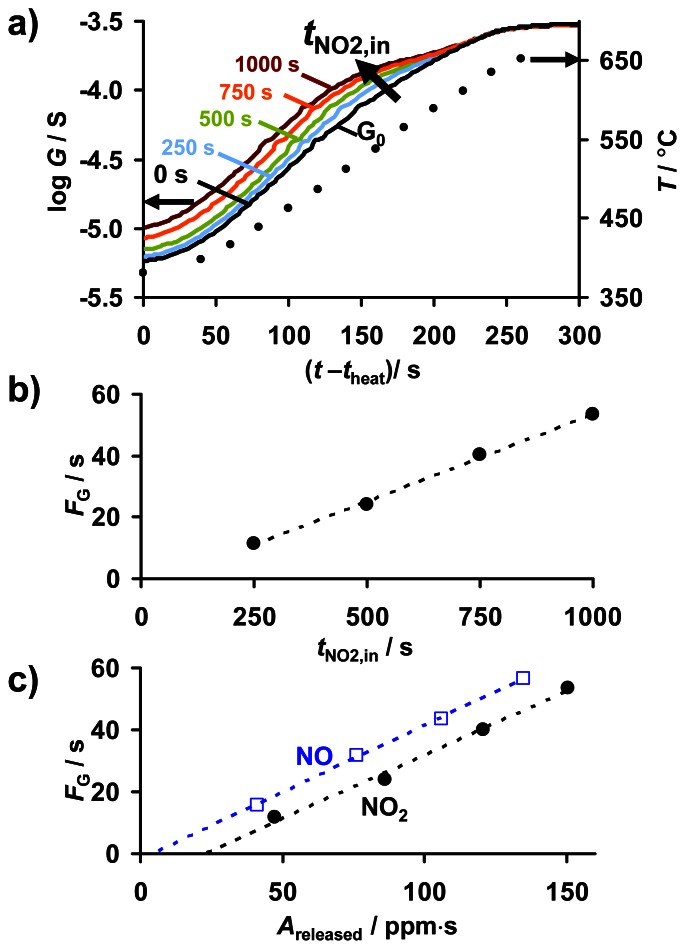
eTPD results after 8 ppm NO_x_ for up to 1,000 s: (**a**) conductance log *G* and temperature *T* during TPD, (**b**) cumulated electrical response *F*_G_ (calculated acc. to Equation (4)) *vs.* the NO_x_ loading time *t*_NO2,in_, (**c**) *F*_G_ as a function of the amount of released NO_x_*A*_released_ for NO and NO_2_ loading, as determined in Figure 9.

The conductance upon releasing NO_x_ provides information about the amount of previously sorbed NO_x_. This may also be useful for NO_x_ dosimetry. Figure 11a depicts the courses of the conductance *G* during thermal regeneration after exposure to 8 ppm NO_2_, for the different loading states indicated by its specific NO_x_ exposure period *t*_NO2,in_. The course of the temperature is shown for comparison (black dots). *G*_0_ reflects the conductance in the NO_x_ unloaded state corresponding to *t*_NO2,in_ = 0.

Being thermally activated, *G*_0_ increases by nearly two orders of magnitude, which agrees with Figure 3. At 380 °C, the conductance in the partly NO_x_ loaded state *G* is higher than *G*_0_. The difference between *G* and *G*_0_ corresponds to the cumulative NO_x_ response, Δ*R*_rel_ (Figure 7). With progressive temperature, log *G* increases like log *G*_0_. The difference between log *G* and log *G*_0_ increases with *t*_NO2,in_, indicating a correlation with the NO_x_ loading level. Between about 480 and 530 °C, the curves of *G* start to converge to those of *G*_0_. Finally, above about 620 °C (230 s) all curves of *G* coincide with *G*_0_ indicating that the unloaded state is recovered. A more detailed analysis reveals that the inflection point in the course of log *G* corresponds to the onset of NO_x_ release shown in Figure 9(a) as *c*_released_. The temperature of the minimum in the slope of log *G* coincides with the temperature of the maximum of *c*_released_. Both are being shifted to lower temperatures, the higher the former loading level was. Hence, the convergence of the curves of log *G* to the reference in the unloaded state can be attributed to thermal NO_x_ release from KMnO_4_/La-Al_2_O_3_, which decreases the temperature-dependent conductivity to the unloaded value.

The comparison of the curves of the conductance *G* during regeneration (Figure 11(a)) suggests that the deviation of the course of log *G* from log *G*_0_ might reflect the amount of previously sorbed NO_x_. In fact, Figure 11(b) reveals a linear correlation between *F*_G_ calculated according to Equation (4) and the preceding sorption interval *t*_NO2,in_. Accounting for the time constant NO and NO_2_ sorption rate in the low loading state (exemplarily shown for NO_2_ in Figure 9(b)), *F*_G_ is also a linear function of *A*_released_ as shown in Figure 11(c) for NO and NO_2_ exposure, respectively. Therefore, besides of Δ*R*_rel_, the cumulated electrical response *F*_G_ of KMnO_4_/La-Al_2_O_3_ during regeneration may also be a suitable sensor signal for the cumulated NO_x_ exposure and the *in-situ* loading level. Again, the resulting sensitivity to NO is slightly higher than those to NO_2_. Furthermore, the sensor response exhibits an offset, in particular, for NO_2_. Besides of the small integration error *A*_offset_ when determining the area under the low level concentration curve during desorption in Figure 9a, these offsets likely originate from NO_2_ adsorption in the feed lines.

**Figure 12.**
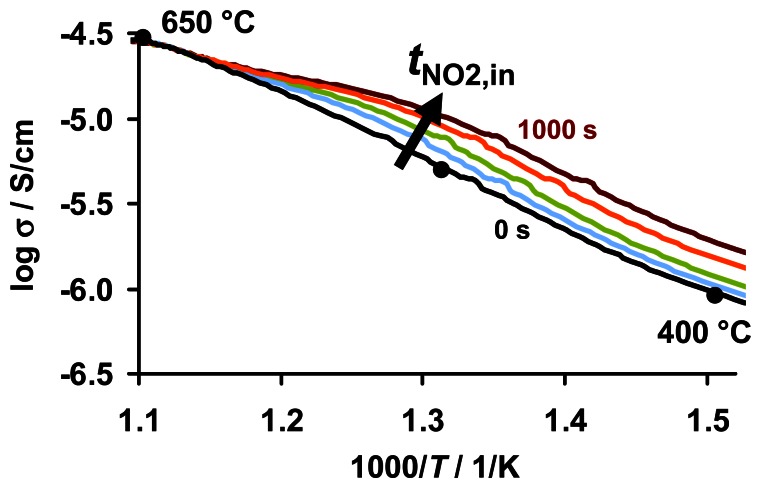
Arrhenius-like plot of the eTPD data in different loading states (lines) compared to the equilibrated unloaded state (black dots).

Considering the electrode geometry, the conductivity σ can be calculated from the conductance and the data from Figure 11(a) can be plotted in an Arrhenius-like representation in the area of a constant heating rate as depicted in Figure 12. The data points of the electrical characterization of the KMnO_4_/La-Al_2_O_3_ sample after equilibration at various temperatures shown in Figure 3(c) are added to Figure 12 as black dots. Concerning the thermally activated conductivity in the unloaded state, the direct comparison reveals that the eTPD data agree well with those obtained in the equilibrated state. This confirms the recovery of the sorption sites by heating to 650 °C. The NO_x_ saturated state of the carbonates was found to give a more pronounced transition in the curve of the equilibrated temperature-dependent conductivity upon thermal decomposition [32].

The investigation of NO_x_ sorption on KMnO_4_/La-Al_2_O_3_ demonstrates that eTPD enables one to correlate the analyte-induced electrical response quantitatively with the actual analyte loading state. This is achieved by observing the electrical properties and the gas desorption characteristics simultaneously. The eTPD method might enhance the understanding of the analyte sorption related electrical properties of functional materials applied for gas sensing or catalysis. Approaches on interpreting the conductivity during thermally releasing gas species are reported in the literature as well, but—to our knowledge—only without a simultaneous quantitative analysis of the desorption peak and hence with a missing correlation with the actual loading state under identical conditions. Colin et al.[50] and Fortin et al.[51] modeled the electrical influence of chemisorbed gases on semiconductors upon heating and verified it for the system oxygen-CdSe. The slope of the conductivity is reported to give information on desorption or binding energies of the species [51–53]. Rossé et al.[53] explains in detail the course of the resistivity in the Arrhenius-like representation during TPD being dependent on the heating rate and the amount of chemisorbed species. Additionally, the recovery of the initial loading state is described as a convergence to the unloaded reference. This description agrees fully with the interpretation of the results on NO_x_ loaded KMnO_4_/La-Al_2_O_3_ in Figure 11. However, applying eTPD, these electrical results were additionally verified by the analysis of the simultaneous desorption peak (Figure 9(a)). Yamazoe et al.[54] and Rodríguez-Gonzáles et al.[55] compared the conductivity with the evolution of desorbed gases as well, but these tests were performed on multiple samples in different setups. The Simon group [55–57] investigated the temperature dependent NH_3_ loading level of zeolites being active for the selective catalytic reduction (SCR) of NO_x_. The conductivity upon heating reveals information on the conduction mechanism, the NH_3_ desorption temperature as well as the SCR active temperature region allowing for *in-situ* reaction monitoring. Kubinski et al.[58] demonstrated that the average resistance during the thermally-induced NH_3_ release from an SCR zeolite catalyst correlates with the former NH_3_ exposure. This enables *in-situ* monitoring of the amount of sorbed NH_3_ with a higher sensitivity compared to those in the NH_3_ sorption mode [58].

**Figure 13.**
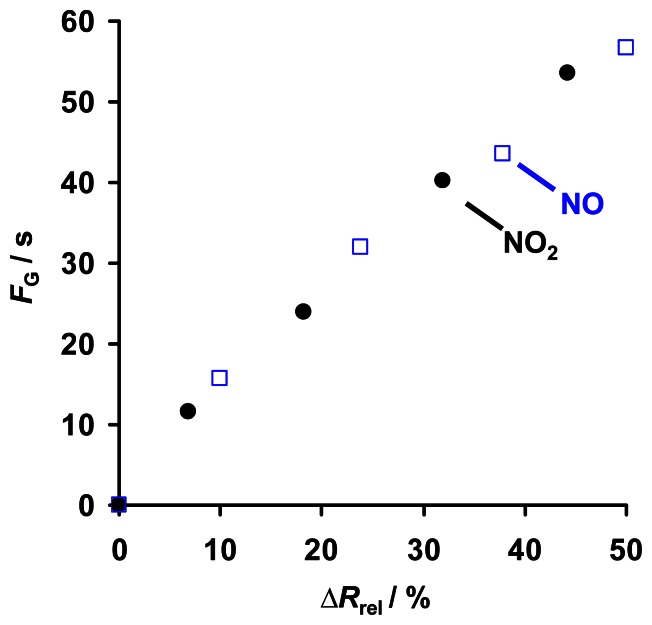
Correlation between electrical responses Δ*R*_rel_ (during sorption) and *F*_G_ (upon regeneration) affected by NO_x_ exposure.

The equivalency of the two conductivity-related sensor responses Δ*R*_rel_ and *F*_G_ of KMnO_4_/La-Al_2_O_3_ as a measure for the cumulated NO_x_ exposure is demonstrated in Figure 13 as a monotone and almost linear correlation, independent of the type of exposed NO_x_ species. Both values can be applied as NO_x_ dosimeter-type sensor responses and correlate linearly with the quantity of sorbed NO_x_, enabling *in-situ* monitoring of the loading state, although they are analyzed upon NO_x_ sorption at 380 °C (Δ*R*_rel_) and upon NO_x_ release by heating up to 650 °C (*F*_G_), respectively. Hence, dependent on the application and the information of interest, two different sensing modes are feasible with the proposed impedimetric NO_x_ dosimeter based on KMnO_4_. In both cases, NO_x_ is accumulated in the sensitive layer at sorption temperature and thermally released during periodic regeneration intervals. However, in the first method, the change in the conductance during NO_x_ accumulation in the low loading state is monitored as a continuous and linear measure for the cumulated NO_x_ exposure as well as for the amount of sorbed NO_x_. Concentration information can be obtained time-continuously from the signal derivative. On the contrary, in the second method, the integrated difference between the conductivity during NO_x_ release upon heating and the conductivity in the unloaded state serves as the measurand. Unfortunately, no time-continuous information on the NO_x_ concentration can be obtained.

Simultaneous NO_x_ detection in the sorption and release mode may be realized on one single sensor platform, with multiple independently heated sensitive layers, as described in [17]. A combination of both sensing modes to extract further information will be the focus of further research. The redundant sensing information is expected to enable a plausibility consideration of the time resolved sensor signal during NO_x_ sorption and of the regeneration success. Additionally, the linear measurement range for the NO_x_ exposure is expected to be enhanced in the regeneration mode. Another important issue of gas sensors is the sensitivity to other gases as well as poisoning of sensitive layers, e.g., by SO_2_ [59,60]. Since interfering gases might affect NO_x_ sorption and release as well as the temperature-dependent conductivity differently, a combination of the electrical responses upon NO_x_ sorption and release might be particularly useful.

## 5. Conclusions

This initial study demonstrates the suitability of decomposed KMnO_4_ deposited on La-stabilized alumina as dosimeter-type sensitive material with two different operation methods and as a NO_x_ sorbent in catalysts with electrical *in-situ* characterization potential. The impedimetric sensor response to low levels of NO and NO_2_ was found to be irreversible under isothermal conditions at 380 °C. These dosimeter-type sensing characteristics are reproducible if sorbed NO_x_ is released by heating up to 650 °C to recover the sorption capacity. The resistance change of KMnO_4_/La-Al_2_O_3_ in the low loaded state correlates linearly with the cumulated NO_x_ exposure, enabling low level NO_x_ detection due to the NO_x_ oxidizing and sorbing capability of the KMnO_4_-based material. The sensor responds slightly more sensitively to NO than to NO_2_.

By combining the electrical response with thermal programmed desorption (eTPD), the change in the electrical properties of KMnO_4_/La-Al_2_O_3_ can be related to the thermally released quantity of NO_x_. This novel method enables the quantitative correlation between the electrical response and the NO_x_ loading in the sensor (or catalyst) material. The amount of NO_x_ sorbed on KMnO_4_/La-Al_2_O_3_, estimated from the released amount, increases linearly with the cumulated NO_x_ exposure (or dose), resulting in a time constant NO_x_ sorption rate. The resistance change during NO_x_ sorption correlates linearly with the amount of sorbed NO_x_ and hence with the NO_x_ exposure. Therefore, information on the NO_x_ concentration can be obtained time-continuously from the signal derivative. Additionally, the thermally activated conductivity of KMnO_4_ is affected by the NO_x_ release upon heating. The deviation from the course of the temperature-dependent conductivity in the unloaded state is another linear measure for the previously stored amount of NO_x_. As a result, NO_x_ exposure and NO_x_ loading dependent electrical response can be analyzed either during NO_x_ sorption or release enabling dosimeter-like NO_x_ sensing or electrical *in-situ* monitoring.

## References

[1] Fergus J.W. (2007). Materials for high temperature electrochemical NO_x_ gas sensors. Sens. Actuators B Chem..

[2] Yamazoe N., Miura N. (1994). Environmental gas sensing. Sens. Actuators B Chem..

[3] Afzal A., Cioffi N., Sabbatini L., Torsi L. (2012). NO_x_sensors based on semiconducting metal oxide nanostructures: Progress and perspectives. Sens. Actuators B Chem..

[4] (2008). Directive 2008/50/EC of the european parliament and of the council of 21 May 2008 on ambient air quality and cleaner air for europe. Off. J. EU.

[5] Twigg M.V. (2007). Progress and future challenges in controlling automotive exhaust gas emissions. Appl. Catal. B.

[6] Groβ A., Beulertz G., Marr I., Kubinski D.J., Visser J.H., Moos R. (2012). Dual mode NO_x_ sensor: Measuring both the accumulated amount and instantaneous level at low concentrations. Sensors.

[7] Matsuguchi M., Kadowaki Y., Tanaka M. (2005). A QCM-based NO_2_ gas detector using morpholine-functional cross-linked copolymer coatings. Sens. Actuators B Chem..

[8] Sasaki D.Y., Singh S., Cox J.D., Pohl P.I. (2001). Fluorescence detection of nitrogen dioxide with perylene/PMMA thin films. Sens. Actuators B Chem..

[9] Tanaka T., Guilleux A., Ohyama T., Maruo Y.Y., Hayashi T. (1999). A ppb-level NO_2_ gas sensor using coloration reactions in porous glass. Sens. Actuators B Chem..

[10] Jung W., Sahner K., Leung A., Tuller H.L. (2009). Acoustic wave-based NO_2_ sensor: Ink-jet printed active layer. Sens. Actuators B Chem..

[11] Geupel A., Schönauer D., Röder-Roith U., Kubinski D.J., Mulla S., Ballinger T.H., Chen H.-Y., Visser J.H., Moos R. (2010). Integrating nitrogen oxide sensor: A novel concept for measuring low concentrations in the exhaust gas. Sens. Actuators B Chem..

[12] Brunet J., Parra Garcia V., Pauly A., Varenne C., Lauron B. (2008). An optimised gas sensor microsystem for accurate and real-time measurement of nitrogen dioxide at ppb level. Sens. Actuators B Chem..

[13] Yamazoe N., Shimanoe K., Aswal D.K., Gupta S.K. (2007). Overview of Gas Sensor Technology. Science and Technology of Chemiresistor Gas Sensors.

[14] Shu J.H., Wikle H.C., Chin B.A. (2010). Passive chemiresistor sensor based on iron (II) phthalocyanine thin films for monitoring of nitrogen dioxide. Sens. Actuators B Chem..

[15] Groβ A., Bishop S.R., Yang D.J., Tuller H.L., Moos R. (2012). The electrical properties of NO_x_-storing carbonates during NO_x_ exposure. Solid State Ionics.

[16] Groβ A., Richter M., Kubinski D.J., Visser J.H., Moos R. (2012). The effect of the thickness of the sensitive layer on the performance of the accumulating NO_x_ sensor. Sensors.

[17] Brandenburg A., Kita J., Groβ A., Moos R. (2013). Novel tube-type LTCC transducers with buried heaters and inner interdigitated electrodes as a platform for gas sensing at various high temperatures. Sens. Actuators B Chem..

[18] Geupel A., Kubinski D.J., Mulla S., Ballinger T.H., Chen H.Y., Visser J.H., Moos R. (2011). Integrating NO_x_ sensor for automotive exhausts—a novel concept. Sens. Lett..

[19] Fruhberger B., Stirling N., Grillo F.G., Ma S., Ruthven D., Lad R.J., Frederick B.G. (2001). Detection and quantification of nitric oxide in human breath using a semiconducting oxide based chemiresistive microsensor. Sens. Actuators B Chem..

[20] Brogren C., Karlsson H.T., Bjerle I. (1997). Absorption of NO in an alkaline solution of KMnO_4_. Chem. Eng. Technol..

[21] Wei Z.-S., Niu H.-J., Ji Y.-F. (2009). Simultaneous removal of SO_2_ and NO_x_ by microwave with potassium permanganate over zeolite. Fuel Process. Technol..

[22] Becerra M.E., Arias N.P., Giraldo O.H., López-Suárez F.E., Illán-Gómez M.J., Bueno-López A. (2011). Soot combustion manganese catalysts prepared by thermal decomposition of KMnO_4_. Appl. Catal. B.

[23] Lesage T., Saussey J., Malo S., Hervieu M., Hedouin C., Blanchard G., Daturi M. (2007). Operando FTIR study of NO_x_ storage over a Pt/K/Mn/Al_2_O_3_-CeO_2_ catalyst. Appl. Catal. B.

[24] Becerra M.-E., Arias N.-P., Giraldo O.-H., López-Suárez F.-E., Illán-Gómez M.-J., Bueno-López A. (2012). Alumina-supported manganese catalysts for soot combustion prepared by thermal decomposition of KMnO_4_. Catalysts.

[25] Boldyrev V.V. (1969). Mechanism of thermal decomposition of potassium permanganate in the solid phase. J. Phys. Chem. Solids.

[26] Boldyrev V.V. (1986). Topochemistry of thermal decompositions of solids. Thermochimica Acta.

[27] Galwey A.K., Brown M.E. (2007). An appreciation of the chemical approach of V. V. Boldyrev to the study of the decomposition of solids. J. Therm. Anal. Calorim..

[28] Kabanov A.A. (1971). The application of electrophysical effects to the study of the thermal decomposition of solids. Russ. Chem. Rev..

[29] Rosseinsky D.R., Tonge J.S. (1982). Electron transfer in solids. Temperature dependence of dielectric relaxation and conductivity in mixed-valence potassium manganate–permanganate. J. Chem. Soc. Faraday Trans..

[30] Kappenstein C., Pirault-Roy L., Guérin M., Wahdan T., Ali A.A., Al-Sagheer F.A., Zaki M.I. (2002). Monopropellant decomposition catalysts: V. Thermal decomposition and reduction of permanganates as models for the preparation of supported MnO_x_ catalysts. Appl. Catal. A.

[31] Schönauer D., Moos R. (2010). Detection of water droplets on exhaust gas sensors. Sens. Actuators B Chem..

[32] Groβ A., Weller T., Tuller H.L., Moos R. (2013). Electrical conductivity study of NO_x_trap materials BaCO_3_and K_2_CO_3_/La-Al_2_O_3_during NO_x_exposure. Sens. Actuators B Chem..

[33] Wu X., Lin F., Wang L., Weng D., Zhou Z. (2011). Preparation methods and thermal stability of Ba-Mn-Ce oxide catalyst for NO_x_-assisted soot oxidation. J. Environ. Sci..

[34] Wu X., Liu S., Lin F., Weng D. (2010). Nitrate storage behavior of Ba/MnO_x_-CeO_2_ catalyst and its activity for soot oxidation with heat transfer limitations. J. Hazard. Mater..

[35] Xiao J.-H., Li X.-H., Deng S., Xu J.-C., Wang L.-F. (2006). The NO_x_ oxidation-storage and tolerance of SO_2_ poison of Mn/Ba/Al_2_O_3_ catalyst. Acta Phys. Chim. Sin..

[36] Xiao J., Li X., Deng S., Wang F., Wang L. (2008). NO_x_ storage-reduction over combined catalyst Mn/Ba/Al_2_O_3_–Pt/Ba/Al_2_O_3_. Catal. Commun..

[37] Beulertz G., Groβ A., Moos R., Kubinski D.J., Visser J.H. (2012). Determining the total amount of NO_x_ in a gas stream – Advances in the accumulating gas sensor principle. Sens. Actuators B Chem..

[38] Gill L.J., Blakeman P.G., Twigg M.V., Walker A.P. (2004). The use of NO_x_ adsorber catalysts on diesel engines. Top. Catal..

[39] Roy S., Baiker A. (2009). NO_x_ storage-reduction catalysis: From mechanism and materials properties to storage-reduction performance. Chem. Rev..

[40] Epling W.S., Campbell L.E., Yezerets A., Currier N.W., Parks J.E. (2004). II. Overview of the fundamental reactions and degradation mechanism of NO_x_ storage/reduction catalysts. Catal. Rev. Sci. Eng..

[41] Fremerey P., Reiβ S., Geupel A., Fischerauer G., Moos R. (2011). Determination of the NO_x_ loading of an automotive lean NO_x_ trap by directly monitoring the electrical properties of the catalyst material itself. Sensors.

[42] Moos R., Zimmermann C., Birkhofer T., Knezevic A., Plog C., Busch M.R., Ried T. Sensor for Directly Determining the State of a NO_x_Storage Catalyst.

[43] Moos R., Wedemann M., Spörl M., Reiβ S., Fischerauer G. (2009). Direct catalyst monitoring by electrical means: An overview on promising novel principles. Top. Catal..

[44] Le Phuc N., Courtois X., Can F., Royer S., Marecot P., Duprez D. (2011). NO_x_ removal efficiency and ammonia selectivity during the NO_x_ storage-reduction process over Pt/BaO(Fe, Mn, Ce)/Al_2_O_3_ model catalysts. Part I: Influence of Fe and Mn addition. Appl. Catal. B.

[45] Bentrup U., Brückner A., Richter M., Fricke R. (2001). NO_x_ adsorption on MnO_2_/NaY composite: An *in situ* FTIR and EPR study. Appl. Catal. B.

[46] Fricke R., Schreier E., Eckelt R., Richter M., Trunschke A. (2004). Non-isothermal NO_x_storage/release over manganese based traps: Mechanistic considerations. Top. Catal..

[47] Kijlstra W.S., Brands D.S., Poels E.K., Bliek A. (1997). Mechanism of the selective catalytic reduction of NO by NH_3_ over MnOx/Al_2_O_3_. J. Catal..

[48] Li W.B., Yang X.F., Chen L.F., Wang J.A. (2009). Adsorption/desorption of NO_x_ on MnO_2_/ZrO_2_ oxides prepared in reverse microemulsions. Catal. Today.

[49] Takeuchi M., Matsumoto S. (2004). NO_x_ storage-reduction catalysts for gasoline engines. Top. Catal..

[50] Colin Y., Fortin B., Raoult F. (1981). Resistance variation of a semiconduction thin film during a thermal desorption. Phys. Status Solidi A.

[51] Fortin B., Larzul H., Lebigot J., Raoult F., Rosse G. (1985). Model for the resistance variation of a thin semiconducting film during temperature- programmed desorption: Application to the O_2_-CdSe system. Thin Solid Films.

[52] Sanjines R., Lévy F., Demarne V., Grisel A. (1990). Some aspects of the interaction of oxygen with polycrystalline SnO_x_ thin films. Sens. Actuators B Chem..

[53] Rossé G., Raoult F., Fortin B. (1984). Regeneration of CdSe thin films after oxygen chemisorption. Thin Solid Films.

[54] Yamazoe N., Fuchigami J., Kishikawa M., Seiyama T. (1979). Interactions of tin oxide surface with O_2_, H_2_O and H_2_. Surf. Sci..

[55] Rodríguez-González L., Rodríguez-Castellón E., Jiménez-López A., Simon U. (2008). Correlation of TPD and impedance measurements on the desorption of NH_3_ from zeolite H-ZSM-5. Solid State Ionics.

[56] Simons T., Simon U. (2012). Zeolites as nanoporous, gas-sensitive materials for *in situ* monitoring of DeNO_x_-SCR. Beilstein J. Nanotechnol..

[57] Simons T., Simon U. (2011). Zeolite H-ZSM-5: A Microporous Proton Conductor for the *in situ*Monitoring of DeNO_x_-SCR. Mater. Res. Soc. Symp. Proc..

[58] Kubinski D.J., Visser J.H. (2008). Sensor and method for determining the ammonia loading of a zeolite SCR catalyst. Sens. Actuators B Chem..

[59] Groβ A., Hanft D., Beulertz G., Marr I., Kubinski D.J., Visser J.H., Moos R. (2012). The effect of SO_2_on the sensitive layer of a NO_x_dosimeter. Sens. Actuators B Chem..

[60] Rettig F., Moos R., Plog C. (2003). Sulfur adsorber for thick-film exhaust gas sensors. Sens. Actuators B Chem..

